# The Seasonal Variation in Bioactive Compounds Content in Juice from Organic and Non-organic Tomatoes

**DOI:** 10.1007/s11130-013-0352-2

**Published:** 2013-04-23

**Authors:** Ewelina Hallmann, Janusz Lipowski, Krystian Marszałek, Ewa Rembiałkowska

**Affiliations:** 1 WULS-SGGW, Faculty of Human Nutrition and Consumer Sciences, Department of Functional, Organic Food and Commodities, Nowoursynowska 159c, 02-776 Warsaw, Poland; 2 Institute of Agricultural and Food Biotechnology, Department of Fruit and Vegetable Product Technology, Rakowiecka 36, Warsaw, Poland

**Keywords:** Organic tomato juice, Non-organic tomato juice, Vitamin C, Carotenoids, Polyphenols

## Abstract

A specific objective of this paper was to evaluate seasonal changes in bioactive compounds level (carotenoids and polyphenols) in juice prepared from organic and non-organic tomatoes in Poland. In the examined tomato juice, the content of dry matter, vitamin C, carotenoids as well as polyphenols (by HPLC method) has been measured. The presented results indicate the impact of the growing system and the year of production on the composition of tomato juice. The organic tomato juice contained significantly more beta-carotene, chlorogenic acid, rutin as well as more total phenolic acids, gallic acid, p-coumaric acid, total flavonoids, quercetin-3-O-glucoside and quercetin in comparison with the non-organic. The tomato juice from 2008 contained significantly more carotenoids and some flavonoids compared to the one produced in 2009, which contained significantly more dry matter, vitamin C, as well as quercetin and it derivatives.

**Electronic supplementary material:**

The online version of this article (doi:10.1007/s11130-013-0352-2) contains supplementary material, which is available to authorized users.

## Introduction

Tomatoes are very important in human nutrition as they contain a lot of bioactive compounds [1–3]. Raw tomatoes in Poland are eaten mostly in summer and autumn, therefore tomato juice is an important substitute for the raw fruits. In Poland tomato juice is very popular among consumers. In 2012, it took the fifth place in the overall structure of juice consumption (5.2 %), following orange juice (25.4 %), carrot juice (22.7 %), apple juice (15.4 %) and grapefruit juice (5.3 %) [4]. Several studies conducted in Poland and around the world have indicated that organic tomatoes have a higher content of biologically active compounds compared to conventional tomatoes [5, 6]. The main reason is nitrogen fertilization, which is completely different in organic system. Nitrogen synthetic fertilizers are forbidden, and only natural fertilizers (*i.e.*, compost, manure and green manure) are used. According to the GDBH theory, when easily soluble nitrogen is abundant in soil, plants grow quicker but produce less biocompounds, such as phenolics [7]. Being a base for the juice, raw tomatoes have been already analyzed and the results have been published [8]. The results indicated that the raw tomatoes contained significantly more bioactive compounds than non-organic ones, therefore one could expect that the juice obtained from organic tomato fruits should also be marked by higher quality parameters [9, 10]. There is lack of data on the quality of juice from tomatoes produced under controlled agro-technical conditions (organic vs. non-organic). The most common topic is the impact of cultivation on raw plant quality, but the impact on the processed products is almost unknown. Therefore, it seemed very important to undertake the studies described. The aim of this paper was to verify the hypotheses that: 1) the juice prepared from organic tomatoes should contain a higher level of bio-compounds compared to the juice made of non-organic tomatoes, and 2) chemical composition of tomato juice will be different depending on the production year.

## Material and Methods

### Tomato Cultivation

The experiment was conducted in the years 2008–2009. The tomato cultivar ‘Rumba’ was selected as commonly used and very suitable for processing. It was grown in two certified organic farms and two non-organic farms, located in the vicinity, to ensure similar climate and soil conditions. The distance between the farms: organic farm no. 1 (Kaszewska Wola) to non-organic farm no. 1 (Kaszewska Wola)-0.7 km; organic farm no. 2 (Radzanów) to non-organic farm no. 2 (Sewerynów)-7.3 km (Electronic Supplementary Material Table 1).

On all farms tomatoes were grown on separate experimental plots, the surface of which was 150 m^2^ (each replication) for organic farms, and 200 m^2^ for non-organic farms respectively. The care was taken to ensure similar agrotechnical conditions in each experimental farm. During the vegetation season, the plants were regularly watered by the means of drip system. Tomato fruits for processing were collected in the same state of ripeness (red-ripe) from all experimental farms. The sampling procedure has been described below in the subsections.

### Preparation of Tomato Juice

In order to obtain juice, 45 kg of tomatoes—with three replications from each organic and non-organic farm (15 kg from each)—were thoroughly washed, sorted out and crushed using the Kenwood robot (see the section ‘Statistical Analysis’). The fruit pulp obtained was heated at 93 °C, without addition of water. Then, the product was percolated through a sieve of 1.2 mm mesh diameter. Hot thick juice was applied to the jars (capacity of 500 ml). The obtained tomato juice was characterized by pH 3.50 for organic and non-organic tomatoes. The processing method described was selected as the most suitable in the light of general processing recommendations in organic sector; the main aim is to maintain the nutritional value of raw material as high as possible [11]. The same processing method was used for both organic and non-organic tomatoes to minimize the processing impact on juice quality.

### Analysis of Dry Matter

Certain amount of tomato juice (1 g) was subjected to a drying process at the following conditions: temperature 105 °C, constant air pressure, time 24 h. After a day, the samples were cooled in a desiccator and weighed, recording the weight loss. This operation was repeated three times to achieve the state of constant weight, Polish Norm (PN-R-04013:1988) [12].

### Vitamin C

The weighed juice sample (500 mg) was extracted in 2 % oxalic acid. The solution was filtered. The filtrate was collected and then titrated with the 2.6-dichlorophenyloindophenol, Polish Norm (PN-A-04019:1998) [13].

### Carotenoids

The examined tomato juice sample was weighed (50 mg), and put into the plastic test tube, having added 0.1 % BHT (butylated-hydroxytoluene) in hexane, magnesium carbonate (5 mg); the samples were incubated in an ultrasonic bath. Then hexane was added (5 ml) and it was incubated in the bath again. The samples were centrifuged at the speed of 3,780 × *g*. From the test tube 1 ml of supernatant was collected and re-centrifuged at the speed of 41,574.4 × *g*. The amount of 900 μl of supernatant was taken for (HPLC) vials and analysed. To determine carotenoids, it was used a high performance liquid chromatograph (HPLC) Shimadzu, consisting of two LC-20 AD pumps, CMB-20A system controller, SIL-20 AC autosampler, UV/VIS SPD-20AV detector, CTD-20 AC oven, and Max-RP 80A column (250 × 4.60 mm). It was selected an isocratic solvent (methanol), flow 1 ml/min. The wavelength used was 450–470 nm. To identify compounds, it was used an external standard in the form of lycopene (Sigma Aldrich) and beta-carotene (Fluka) with purity of 99.98 % [14].

### Polyphenols

A weighed (100 mg) amount of tomato juice was put into a plastic test tube, and then methanol and 1 % ascorbic acid were added, mixed thoroughly by vortex and incubated in an ultrasonic bath. Then the samples were centrifuged at the speed of 3,780 × *g*. From the test tube 1 ml of extract was collected and re-centrifuged at the speed of 41,574.4 × *g*. The amount of 500 μl of extract was taken for HPLC vials and analysed. For the analysis of phenolic compounds it was used a Synergi Fusion-RP 80i column (250 × 4.60 mm). The gradient flow was applied along with two mobile phases—acetonitrile/deionized water (solvent A 10 % and solvent B 55 %), pH 3; the used gradient program was: 0–21 min. 95 % solvent A and 5 % solvent B; 22–25 min. 50 % solvent A and 50 % solvent B; 26–27 min. 20 % solvent A and 80 % solvent B; 28–32 min. 20 % solvent A and 80 % solvent B; 32–36 min. 95 % solvent A and 5 % solvent B. Time of the analysis 36 min, flow 1 ml/min, wavelength 250–370 nm. Compounds were identified based on Fluka and Sigma Aldrich (Poland) external standards such as: gallic acid, chlorogenic acid, p-coumaric acid, quercetin-3-O-rutinoside, quercetin-3-O-glucoside, quercetin and kaempferol, with purity of 99.5 %.

### Statistical Analysis

There were analysed two organic and two non-organic farms. From every farm three big samples of tomatoes (15 kg each) were taken for analysis. Every sample of 15 kg was converted into tomato juice; three glass jars of tomato juice were produced. Every jar was treated as a laboratory sample. It amounted to 18 samples of tomato juice from each farming system in every year; in total, 72 samples were analysed (in 2008, 18 samples from ORG system and 18 samples from non-organic system, and in 2009, 18 samples from organic system and 18 samples from NON-ORG system). For statistical calculations it selected two-way analysis of variance with the use of the Tukey’s test (α = 0.05) as relevant for both double and multiple comparisons. Lack of statistically significant differences between the examined groups was determined with the same letters. A standard deviation was given at the mean value. All results for the content of the examined compounds are specified in fresh matter of tomato juice.

## Results and Discussion

### Dry Matter Content

The results show that dry matter content in tomato juice from both production systems was very similar (5.66 and 5.63 g/100 g fresh weight (fw), respectively). On the other hand, the tomato juice from 2009 contained a significantly higher level of dry matter in comparison with the first year of the experiment (2008) (Table 2).

The dry matter content in tomato juice is mainly dependent on the content of dry matter in fresh tomato fruits, technological factors as well as the method and time of fruit pulp heating during juice preparation. As reported by Hallmann and Rembiałkowska [10], organic tomato fruits were characterized by a significantly higher content of dry matter, and the juice produced from these fruits contained less water (8.31 g/100 g fw) compared to the non-organic tomato juice (6.19 g/100 g fw). Sánchez-Moreno et al. [15] showed that conventional tomato juice contained only 4.97 g/100 g fw of dry matter (only of dry matter). The lower dry matter content in conventional tomato fruits may be explained by the phenomenon of ‘water swelling’. In a conventional environment plants receive easily soluble mineral fertilizers. They are soil-applied, dissolved in soil solution and along with water absorbed by plant roots. Then, by ascending, *i.e.*, xylem, they are fed into the leaves and fruit [16].

### Vitamin C Content

In the experiment presented, the level of vitamin C was dependent on the juice origin. The non-organic tomato juice was characterized by a significantly higher content of vitamin C (19.30 mg/100 g fw) in comparison with the organic juice (16.80 mg/100 g fw), and the differences were statistically significant (Table 2). These results are inconsistent with most results on raw plants, according to which the level of vitamin C is on average significantly higher in organic plants than non-organic ones [17]. This phenomenon can be explained by the fact that the level of vitamin C increases if the plant is grown under low nitrogen availability conditions [18]. The highest level of vitamin C was found in the juice made in 2009 compared to the one produced in 2008, and the differences were statistically significant. The vitamin C level in tomato juice is affected by the vitamin C content in fresh tomato fruits as well as technological preparation of tomato pulp. It was reported by Barrett et al. [19] that organic fresh tomatoes contained 28.21 mg/100 g fw, while the conventional ones had 47.24 mg/100 g fw. Having heated the tomato pulp, the level of vitamin C in organic tomato pulp decreased to 17.52 mg/100 g fw, and in the conventional fruit to 18.59 mg/100 g fw. Technological processing always causes a decrease of vitamin C in the final product. It is probably caused by the sensitivity of vitamin C to high temperatures during juice production (heating of tomato pulp). Therefore, pulp heating should be as short as possible. Some studies indicate that the use of lower temperature during pulp heating leads to a higher content of vitamin C in tomato juice (11.68 mg/100 g fw compared to 9.07 mg/100 g fw) accordingly [20]. In the experiment presented by Stone et al. [21] freshly prepared tomato pulp contained 17.13 mg/100 g fw of vitamin C, and after heating the level of vitamin C decreased to 13.91 mg/100 g fw. [21]. Another important factor which helps to maintain the vitamin C content in tomato juice is lowering the pH (acidification) of juice. According to Saar and Tsai [22], maintaining the juice pH close to 2 had a stabilizing effect on the vitamin C content in tomato juice (27.9 mg/100 g fw), while in the fresh juice (pH 4.5) the vitamin C content was 10.97 mg/100 g fw and in the slightly acidified juice (pH 3) it was equal to 18.65 mg/100 g fw.

### Carotenoids (Lycopene) Content

The content of lycopene in the tomato juice tested was largely dependent on the juice origin. The organic tomato juice contained significantly less lycopene compared to the non-organic tomato juice, it was 12.52 and 15.43 mg/100 g fw, respectively (Table 2). During the two years of the experiment, the differences in lycopene content in tomato juice were observed. In 2008, the tomato juice contained significantly more lycopene (14.80 mg/100 g fw) in comparison with the 2009 tomato juice (13.15 mg/100 g fw) (Table 1). Similar results were presented by Pieper and Barrett [9], they showed that the content of lycopene in organic tomato sauce was significantly lower (10.29 mg/100 g fw) compared to the conventional one (11.57 mg/100 g fw). At the same time, the authors pointed out the impact of tomato seasonal growing on the lycopene content in tomato sauce. In 2006, the tomato sauce contained more lycopene (11.28 mg/100 g fw) in comparison with 2007 (10.58 mg/100 g fw), but the differences were not statistically significant. The higher lycopene content, the more pink and red tomato juice is. However, the more beta-carotene, the more juice gets darker and takes brown and red shades. Therefore, high content of beta-carotene in tomato juice, despite its antioxidant properties, is undesirable as it adversely affects consumers’ choice. In fresh tomato fruits lycopene is present in *all-trans* configuration, and in this form it is very poorly absorbed by the body. However, after thermal processing of fruits the lycopene configuration changes to *all-cis*, and in this form it is much better available to the human body [23]. The content of lycopene in tomato juice mainly depends on its content in fruits, as well as the method of tomato cultivation and thermal conditions during processing of the product [24]. Similar results were presented in another experiment. Odriozola-Serrano et al. [20] reported that the content of lycopene in tomato juice increases along with rising temperature during the juice preparation. Lycopene is the major carotenoid found in tomatoes; it accounts for over 85 % of all carotenoids detected. In the second place in the carotenoids ranking is beta-carotene, but it represents only 15 % of all carotenoids. In tomato fruits it occurs in very low concentrations, *i.e.*, 0.32–1.23 mg/100 fw in organic tomatoes and 0.29–0.89 mg/100 g fw in conventional ones. The content of lycopene in tomato fruits depends on many factors, including the level of nitrogen in soil. The increase in lycopene content along with more intense nitrogen fertilization is justified by Lacatus et al. [25] as follows: nitrogen is the main element that forms Acetyl-CoA enzyme which plays a central role in the synthesis of carotenoid pigments and converts beta-carotene into lycopene. Dadomo et al. [26] found that with the increased dose of nitrogen the yield of red and uniformly stained fruits as well as the number of fruits per unit of cultivation area were higher.

### Carotenoids (Beta-carotene) Content

The organic tomato juice contained significantly more beta-carotene (0.23 mg/100 g fw) compared to the non-organic tomato juice (0.20 mg/100 g fw). There were also significant differences between the years of the experiment. The juice prepared in 2008 contained more beta-carotene (0.27 mg/100 g fw) compared to the juice from 2009 (0.16 mg/100 g fw) (Table 2). The content of beta-carotene in tomato juice depended on the quality of fresh tomatoes used for juice production and the fruits origin. As reported by Caris-Veyrat et al. [1], organic tomatoes contained significantly more beta-carotene (1.23 mg/100 g fw) compared to conventional ones (0.87 mg/100 g fw). In the experiment presented by Hallmann and Rembiałkowska [5] organic tomatoes also contained significantly more beta-carotene (1.32 mg/100 g fw) compared to conventional ones (0.67 mg/100 g fw). The results are opposite to those presented by Kaack et al. [18], where beta-carotene level was significantly higher in non-organic carrots compared to organic ones. The impact of the cultivation system on beta-carotene level in crops is unclear. Some authors indicate that this pigment is more abundant in the optimal conditions for plant growth. The seasonal variation of beta-carotene content in tomato juice could result from different carotenoid content in tomato fruits. As reported by Hallmann [8], in 2008 tomato fruits contained 0.26 mg/100 g fw of beta-carotene, while in 2009 it was 0.21 mg/100 g fw.

### Phenolic Compounds (Phenolic Acids) Content

The content of total phenolic acids was not significantly higher (51.74 mg/100 g fw) in the organic tomato juice compared to the non-organic one (45.35 mg/100 g fw). The organic tomato juice also contained a higher level of individual phenolic acids: gallic, chlorogenic and p-coumaric, but only in the case of chlorogenic acid content the difference was statistically significant (Table 3).

The results are consistent with the previous results presented by many authors, which indicate that organic plants contained more phenolic compounds than the non-organic ones. It can be explained as follows: the lower nitrogen availability in soil mostly results in the increased content of phenolic compounds [7]. There was not observed any seasonal variation in total and individual phenolic acid content in the tomato juice. The variation between the experimental years was recorded only in the case of chlorogenic acid. The juice from 2009 contained significantly more chlorogenic acid (2.75 mg/100 g fw) compared to the first year (1.67 mg/100 g fw) (Table 3). The content of phenolic compounds in tomato juice is affected by many factors, *e.g.*, tomato variety, method of cultivation and temperature of tomato pulp heating. In tomato juice phenolic acids are the main polyphenol pool. As reported by Podsędek et al. [27], the mean content of total polyphenols in tomato juice ranges from 26.77 to 52.26 mg/100 g fw. Odriozola-Serrano et al. [20] reported that the temperature during juice preparation had no effect on the total phenols content in the samples of tomato juice. The use of lower temperature (60 °C), instead of the higher one (90 °C), showed that the juice contained a similar level of phenolic compounds, *i.e.*, 90.0 mg/100 g fw. Another factor affecting the content of total polyphenols in tomato juice might be acidity. According to Saar and Tsai [22], in acidified tomato juice (pH 2) the total polyphenol content was 50.40 mg/100 g fw, while in the fresh juice (pH 4.50), it was much lower and amounted to 38.53 mg/100 g fw. In the experiment presented by Vallverdú-Queralt et al. [28], the level of polyphenols in the tomato juice purchased in shops was much lower in conventional product than the organic one (9.28–10.55  and 11.34–12.89 mg/100 fw).

### Phenolic Compounds (Flavonoids) Content

The total flavonoid content was significantly higher in the organic tomato juice (2.81 mg/100 g fw) compared to the non-organic one (2.57 mg/100 g fw). The organic tomato juice contained significantly more individual flavonoids: quercetin 3-O-rutinoside and quercetin compared to the non-organic tomato juice (Table 4).

In both experimental years, it was observed a different level of total flavonoids and individual flavonoids between the tomato juices examined. In 2009, the tomato juice contained significantly more total flavonoids, quercetin-3-O-rutinoside, quercetin-3-O-glucoside and quercetin (no kaempferol) compared to the juice from 2008 (Table 3). It seems that the main flavonoid which occurred in the tomato juice is quercetin and its derivatives (as rutinoside and glucoside). According to Odriozola-Serrano et al. [20], the conventional tomato juice contained 1.97 mg/100 g fw of quercetin. The results presented are similar to the studies by Pieper and Barrett [9], who obtained the value of 1.06 mg/100 g fw for the organic tomato juice, while in the case of the conventional one it was 0.82 mg/100 g fw.

### The Impact of the Year

The impact of the year on the tomato juice composition was stronger than the impact of cultivation method, and in most cases the differences were statistically significant (Tables 2–4). Considering the 15 parameters measured, in 10 cases out of 15 the effect of the year was significant, and only in 6 cases out of 15 the cultivation was considered.

## Conclusions

The studies presented indicate that the cultivation system of tomato had a significant impact on bioactive compounds content in tomato juice. The non-organic tomato juice contained less dry matter, kaempferol and significantly more vitamin C as well as lycopene, as compared with the organic one. The organic tomato juice contained significantly more beta-carotene, chlorogenic acid, quercetin-3-O-rutinoside as well as more total and individual phenolic acids (gallic acid and p-coumaric acid), total and individual flavonoids (quercetin-3-O-glucoside and quercetin) in comparison with the non-organic one. In the two experimental years, the effect of growing season was observed. The tomato juice from 2008 contained significantly more lycopene, beta-carotene and kaempferol in comparison with the juice produced in 2009, when the samples contained significantly more dry matter, vitamin C, chlorogenic acid, total flavonoids, quercetin-3-O-rutinoside, quercetin-3-O-glucoside as well as quercetin. The impact of the year on the composition of tomato juice was stronger than the impact of the cultivation system.

## Electronic Supplementary Material

Below is the link to the electronic supplementary material.

ESM 1 (PDF 113 kb)

ESM 2 (PDF 88 kb)

ESM 3 (PDF 88 kb)

ESM 4 (PDF 91 kb)
