# Supplementary material for: The Seasonal Variation in Bioactive Compounds Content in Juice from Organic and Non-organic Tomatoes
Source: Plant Foods Hum Nutr. 2013 Apr 23;68(2):171–6. doi: 10.1007/s11130-013-0352-2 (PMC3659276; doi:10.1007/s11130-013-0352-2)
Supplement: Supplementary file 1 — (PDF 113 kb) [file 11130_2013_352_MOESM1_ESM.pdf]

attachment to manuscript
Click here to download attachment to manuscript: 2. ESM_Hallmann et al_ 20 III 2013.doc
Click here to view linked References

Tab 1. Characteristic of localization, fertilizers regime and plant protection used for organic and conventional cultivation of tomatoes (average value for 2008-2009)

| | | | | | fertilization range (kg ha$^{-1}$) | | | | | |
|---|---|---|---|---|---|---|---|---|---|---|
| Farm | Localization | Type of soil | Fertilizers | Plant protection system | N | P | K | Mg | Ca | Micro elements |
| organic farm no.1 | Kaszewska Wola /51°30' N 20°55' E/ | sandy- loamy, middle soil III category 40% (floatable particles) pH 7.2 | green manure (50t ha$^{-1}$), Patentkali (P:S 30:10) basaltic flour, Humobak (mixture of bacteria and soil fungi) | Bioczos BR (garlic extrakt), Antifung 20 SL | 140 | 30 | 205 | 90 | 130 | yes |
| organic farm no.2 | Radzanów /51°56' N 20°05' E/ | sandy- loamy, slight soil II category 35% (floatable particles) pH 7.1 | compost 60%, Biohumus | Grevit 200 SL | 180 | 60 | 140 | 90 | 160 | yes |
| non-organic farm no.1 | Kaszewska Wola /51°19' N 20°58' E/ | sandy-clay, slight soil II category 20% (floatable particles) pH 7.0 | cow manure (15t ha$^{-1}$), organic chalk, calcium and magnesium nitrate | Sumilex 500 SC | 220 | 100 | 170 | 60 | 220 | no |
| non-organic farm no.2 | Sewerynów /51°28' N 20°54' E/ | sandy- loamy, slight soil II category 15% (floatable particles) pH 6.9 | Polidap (N:P:S 18:46:5), Grandar (N:P:K 3:9:19)., Nitrosofosphate | Mospilan 20 SP (acetamipiryde) Betoksan S 050 SL (2-naftoxyacetic acid) | 300 | 210 | 140 | 35 | 480 | yes |
