# Supplementary material for: The Seasonal Variation in Bioactive Compounds Content in Juice from Organic and Non-organic Tomatoes
Source: Plant Foods Hum Nutr. 2013 Apr 23;68(2):171–6. doi: 10.1007/s11130-013-0352-2 (PMC3659276; doi:10.1007/s11130-013-0352-2)
Supplement: Supplementary file 2 — (PDF 88 kb) [file 11130_2013_352_MOESM2_ESM.pdf]

Tab. 2. The content of dry matter (g/100 g fw), vitamin C and carotenoids (mg/100 g fw) in organic and conventional produced tomatoes juice in two years of experiment (average value ± standard deviation)

**2008**	dry matter	vitamin C	lycopene	beta-carotene
Org.1	5.47±0.29	14.79±0.14	11.54±0.68	0.34±0.02
Org.2	5.92±0.03	11.55±0.91	12.51±0.20	0.25±0.01
Org.3	5.98±0.04	10.84±0.11	14.12±0.11	0.22±0.01
Org.4	4.32±0.01	11.85±0.31	17.23±0.88	0.23±0.01
Org.5	5.31±0.04	13.93±0.29	14.03±0.39	0.39±0.01
Org.6	5.16±0.05	12.71±1.33	12.60±±0.35	0.25±0.03
Non-org.1	4.60±0.02	9.74±0.08	17.98±0.08	0.19±0.01
Non-org.2	6.95±0.03	14.77±0.77	14.20±0.09	0.18±0.01
Non-org.3	4.37±0.01	8.21±0.12	15.14±0.07	0.27±0.01
Non-org.4	6.31±0.04	14.03±0.28	16.43±0.36	0.11±0.01
Non-org.5	5.08±0.03	13.06±0.59	16.47±0.22	0.42±0.02
Non-org.6	5.90±0.03	13.81±0.45	15.31±0.30	0.36±0.01
**2009**				
Org.1	5.57±0.32	24.57±0.28	10.18±0.09	0.15±0.01
Org.2	6.55±0.34	17.73±0.77	11.47±0.34	0.21±0.01
Org.3	5.43±0.41	23.34±0.12	12.45±1.18	0.20±0.01
Org.4	4.55±0.08	11.63±0.28	14.50±0.29	0.12±0.02
Org.5	6.33±0.08	24.23±0.59	10.35±0.10	0.26±0.02
Org.6	7.31±0.12	24.41±0.23	9.26±0.17	0.19±0.02
Non-org.1	5.93±0.03	25.22±0.10	14.62±0.02	0.09±0.01
Non-org.2	5.44±0.03	24.09±0.53	15.34±0.10	0.10±0.01
Non-org.3	5.25±0.06	27.29±0.30	18.31±0.08	0.10±0.01
Non-org.4	5.46±0.11	24.64±0.87	14.15±0.83	0.09±0.02
Non-org.5	6.21±0.01	30.75±0.21	12.89±0.77	0.24±0.01
Non-org.6	6.13±0.10	26.01±0.57	14.33±0.34	0.23±0.01
mean Org. juices	5.66±1.02 a*	16.80±5.56 a	12.52±2.18 a	0.23±0.07 b
mean Non-org. juices	5.63±0.72 a	19.30±7.42 b	15.43±1.59 b	0.20±0.11 a
mean tomatoes juices (2008)	5.44±0.91 A	12.44±2.06 A	14.80±1.95 B	0.27±0.09 B
mean tomatoes juices (2009)	5.85±0.71 B	23.66±4.68 B	13.15± 2.52 A	0.16±0.06 A
**p-value**				
production system	n.s.**	0.0013	0.0001	0.049
year	0.0002	<0.0001	<0.0001	<0.0001
production x year	0.005	0.0003	n.s.	n.s.

* means in a columns followed by the different letter are significantly different at the 5% level of probability (α=0.05) by Tukey's test; ** not significant statistically (α>0.05)
small letter focused differences between juice production system, CAPITAL letter focused differences between experimental years
