# Supplementary material for: The Seasonal Variation in Bioactive Compounds Content in Juice from Organic and Non-organic Tomatoes
Source: Plant Foods Hum Nutr. 2013 Apr 23;68(2):171–6. doi: 10.1007/s11130-013-0352-2 (PMC3659276; doi:10.1007/s11130-013-0352-2)
Supplement: Supplementary file 3 — (PDF 88 kb) [file 11130_2013_352_MOESM3_ESM.pdf]

Tab. 3. The content of total phenolic acids and individual phenolic acids (mg/100 g fw) in organic and conventional produced tomatoes juice in two years of experiment (average value ± standard deviation)

**2008**	total phenolic acids	gallic acid	chlorogenic acid	p-coumaric acid
Org.1	47.56±2.53	45.54±2.41	1.91±0.12	0.117±0.01
Org.2	30.21±2.43	28.49±2.34	1.58±0.09	0.145±0.01
Org.3	33.63±4.01	31.74±3.38	1.69±0.18	0.208±0.01
Org.4	66.04±1.93	64.86±1.92	1.07±0.01	0.107±0.02
Org.5	79.28±1.35	75.40±1.34	3.77±0.02	0.108±0.02
Org.6	68.27±1.87	66.11±1.81	2.03±0.01	0.138±0.01
Non-org.1	41.26±0.81	40.39±0.80	0.77±0.01	0.102±0.01
Non-org.2	70.86±1.34	69.66±1.42	1.09±0.01	0.118±0.07
Non-org.3	24.60±0.11	23.39±0.11	1.10±0.01	0.110±0.01
Non-org.4	56.91±0.35	55.69±0.36	1.06±0.01	0.159±0.01
Non-org.5	40.97±0.16	38.06±0.14	2.72±0.02	0.190±0.02
Non-org.6	42.02±0.22	40.64±0.20	1.26±0.02	0.118±0.01
**2009**				
Org.1	49.30±3.01	46.07±2.96	3.10±0.10	0.130±0.01
Org.2	38.21±0.80	34.52±0.80	3.55±0.17	0.136±0.01
Org.3	40.94±3.68	37.21±3.59	3.59±0.27	0.142±0.02
Org.4	68.41±1.37	66.24±1.36	2.04±0.04	0.141±0.01
Org.5	53.87±0.52	50.72±0.49	3.04±0.03	0.105±0.01
Org.6	45.14±1.06	42.51±1.01	2.51±0.05	0.124±0.01
Non-org.1	62.51±1.14	59.77±1.13	2.63±0.02	0.117±0.01
Non-org.2	53.70±0.33	51.69±0.29	1.88±0.01	0.129±0.04
Non-org.3	29.63±0.89	26.71±0.87	2.82±0.09	0.103±0.01
Non-org.4	58.34±0.15	55.51±0.22	2.70±0.12	0.130±0.02
Non-org.5	31.40±5.06	28.60±5.07	2.68±0.01	0.127±0.01
Non-org.6	31.94±9.44	29.31±9.45	2.51±0.08	0.119±0.01
mean Org. juices	51.74±15.11 a	49.12±15.08 a	2.49±0.87 b	0.134±0.03 a
mean Non-org. juices	45.35±14.54 a	43.29±14.68 a	1.93±0.78 a	0.127±0.04 a
mean tomatoes juices (2008)	50.14±17.09 A	48.33±16.82 A	1.67±0.82 A	0.135±0.04 A
mean tomatoes juices(2009)	46.95±12.77 A	44.07±12.96 A	2.75±0.51 B	0.125±0.02 A
**p-value**				
production system	n.s.**	n.s.	0.0005	n.s.
year	n.s.	n.s.	<0.0001	n.s.
production x year	n.s.	n.s.	n.s.	n.s.

* means in a columns followed by the different letter are significantly different at the 5% level of probability (α=0.05) by Tukey's test; ** not significant statistically (α>0.05)

small letter focused differences between juice production system, CAPITAL letter focused differences between experimental years
