# Supplementary material for: The Seasonal Variation in Bioactive Compounds Content in Juice from Organic and Non-organic Tomatoes
Source: Plant Foods Hum Nutr. 2013 Apr 23;68(2):171–6. doi: 10.1007/s11130-013-0352-2 (PMC3659276; doi:10.1007/s11130-013-0352-2)
Supplement: Supplementary file 4 — (PDF 91 kb) [file 11130_2013_352_MOESM4_ESM.pdf]

Tab.4. The content of total flavonoids and individual flavonoids (mg/100 g fw) in organic and conventional produced tomatoes juice in two years of experiment (average value ± standard deviation)

**2008**	total flavonoids	quercetin-3-O-rutinoside	quercetin-3-O-glucoside	quercetin	kaempferol
Org.1	1.98±0.04	0.46±0.02	0.21±0.01	0.67±0.02	0.64±0.04
Org.2	2.21±0.15	0.56±0.03	0.23±0.01	0.75±0.02	0.68±0.14
Org.3	2.41±0.06	0.64±0.01	0.22±0.01	0.80±0.03	0.75±0.07
Org.4	2.10±0.05	0.52±0.01	0.11±0.01	0.92±0.03	0.55±0.01
Org.5	2.07±0.11	0.77±0.01	0.13±0.01	0.79±0.04	0.39±0.03
Org.6	1.40±0.10	0.26±0.02	0.11±0.01	0.57±0.09	0.46±0.01
Non-org.1	1.19±0.03	0.18±0.01	0.07±0.01	0.46±0.01	0.48±0.02
Non-org.2	1.61±0.02	0.33±0.01	0.14±0.01	0.69±0.01	0.46±0.02
Non-org.3	1.18±0.01	0.15±0.02	0.05±0.01	0.44±0.02	0.54±0.01
Non-org.4	1.85±0.02	0.53±0.01	0.12±0.01	0.62±0.01	0.57±0.01
Non-org.5	1.86±0.02	0.57±0.01	0.12±0.01	0.50±0.01	0.67±0.01
Non-org.6	1.43±0.01	0.23±0.01	0.11±0.02	0.58±0.01	0.51±0.02
**2009**					
Org.1	3.06±0.15	1.24±0.02	0.32±0.02	1.28±0.02	0.22±0.02
Org.2	3.69±0.18	1.78±0.07	0.32±0.01	1.35±0.04	0.22±0.01
Org.3	3.63±0.23	1.83±0.11	0.37±0.06	1.19±0.03	0.24±0.02
Org.4	2.99±0.03	1.62±0.08	0.22±0.02	0.99±0.01	0.16±0.01
Org.5	3.91±0.07	1.55±0.01	0.62±0.01	1.38±0.01	0.36±0.01
Org.6	4.27±0.11	1.69±0.04	0.60±0.01	1.59±0.01	0.38±0.01
Non-org.1	3.37±0.02	1.47±0.01	0.31±0.01	1.29±0.01	0.30±0.01
Non-org.2	3.33±0.02	1.57±0.01	0.35±0.01	1.18±0.01	0.24±0.01
Non-org.3	3.30±0.04	1.63±0.03	0.28±0.01	1.15±0.02	0.23±0.01
Non-org.4	2.83±0.37	1.37±0.41	0.26±0.02	0.98±0.1	0.22±0.02
Non-org.5	4.33±0.34	1.62±0.38	0.80±0.02	1.57±0.01	0.35±0.01
Non-org.6	4.52±0.14	1.82±0.10	0.63±0.01	1.34±0.03	0.72±0.02
mean Org. juices	2.81±0.88 a	1.08±0.27 b	0.29±0.17 a	1.02±0.02 b	0.42±0.20 a
menan Non-org. juices	2.57±1.16 a	0.96±0.16 a	0.27±0.22 a	0.90±0.08 a	0.44±0.17 a
mean tomatoes juices (2008)	1.77±0.39 A	0.43±0.09 A	0.13±0.05 A	0.65±0.05 A	0.56±0.12 B
mean tomatoes juices (2009)	3.60±0.56 B	1.60±0.24 B	0.42±0.18 B	1.28±0.09 B	0.30±0.14 A
**p-value**					
production system	n.s.**	0.0171	n.s.	0.014	n.s.
year	<0.0001	<0.0001	<0.0001	<0.0001	<0.0001
production x year	n.s.	n.s.	n.s.	0.043	n.s.

* means in a columns followed by the different letter are significantly different at the 5% level of probability (α=0.05) by Tukey's test; ** not significant statistically (α>0.05) small letter focused differences between juice production system, CAPITAL letter focused differences between experimental years
